# Supplementary material for: Prion protein conversion at two distinct cellular sites precedes fibrillisation
Source: Nat Commun. 2023 Dec 15;14:8354. doi: 10.1038/s41467-023-43961-1 (PMC10724300; doi:10.1038/s41467-023-43961-1)
Supplement: Supplementary file 1 — Supplementary Information [file 41467_2023_43961_MOESM1_ESM.pdf]

Supplementary Information

Prion protein conversion at two distinct cellular sites precedes fibrillisation

Juan Manuel Ribes[1#], Mitali P. Patel[1#], Hazim A. Halim[1], Antonio Berretta[1], Sharon A. Tooze[2], Peter-Christian Klöhn[1*]

[1]Medical Research Council Prion Unit at UCL, Institute of Prion Diseases, University College London, London W1W 7FF, United Kingdom

[2]Molecular Cell Biology of Autophagy Laboratory, the Francis Crick Institute, London NW1 1BF, United Kingdom

[#] These authors contributed equally

Correspondence: p.kloehn@prion.ucl.ac.uk

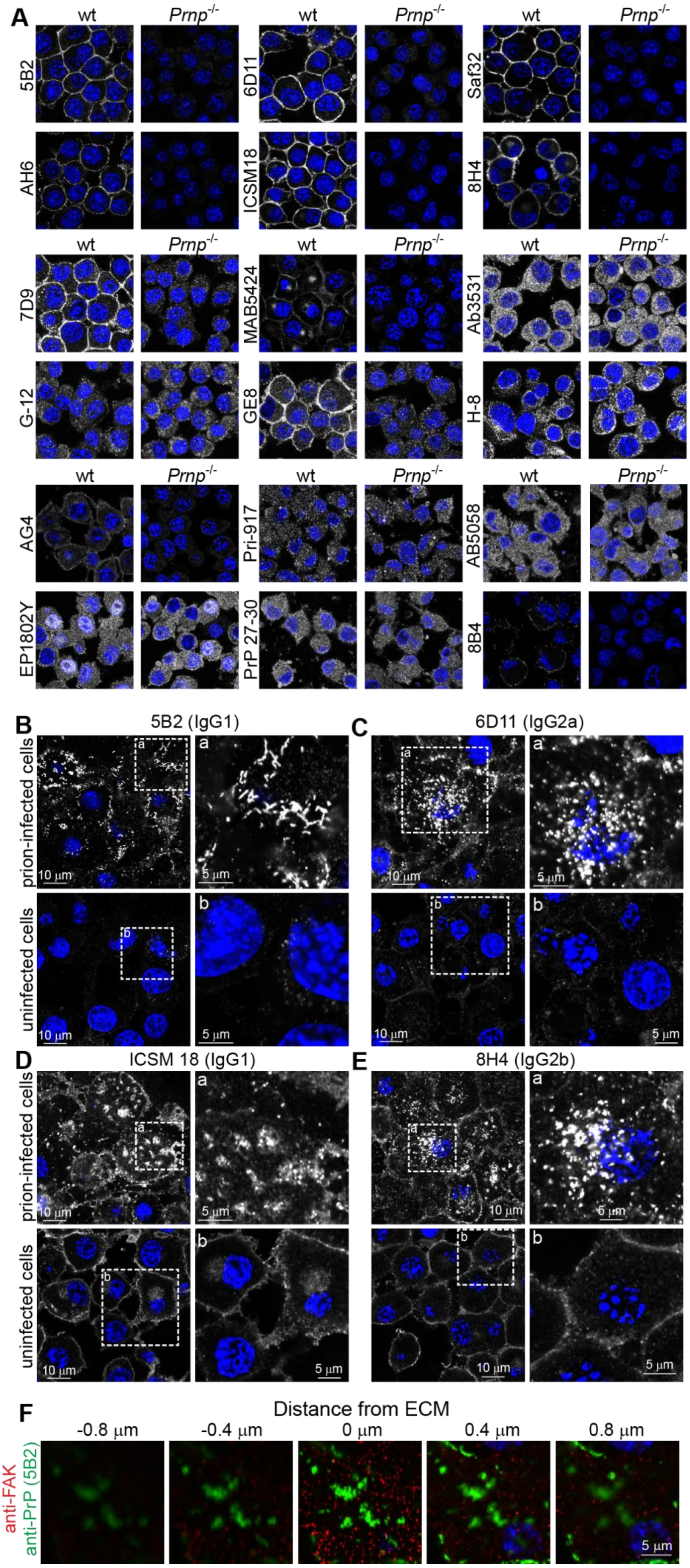
A
wt
Prnp-/-
5B2
6D11
Saf32
AH6
ICSM18
8H4
7D9
MAB5424
Ab3531
G-12
GE8
H-8
AG4
Pri-917
AB5058
EP1802Y
PrP 27-30
8B4
B
5B2 (IgG1)
C
6D11 (IgG2a)
prion-infected cells
uninfected cells
10 μm
5 μm
D
ICSM 18 (IgG1)
E
8H4 (IgG2b)
F
Distance from ECM
-0.8 μm
-0.4 μm
0 μm
0.4 μm
0.8 μm
anti-FAK
anti-PrP (5B2)

Supplementary Fig. 1: Anti-PrP antibody validation and PrP^d phenotypes

(A) To assess the target specificity of anti-PrP antibodies, *Prnp* was deleted in N_2a cells by CRISPR-Cas9 as specified in Methods. Aliquots of N_2a (wt) and N_2a-*Prnp*$^{-/-}$ (*Prnp*-ko) cells were plated into wells of 8-well chamber slides at a concentration of 5 x 10^4 cells/ml OFCS. After three days of culture, cells were fixed with 3.7% formalin for 12 minutes, washed once with PBS and permeabilised with 0.04% Triton X-100 for 10 minutes. Cells were incubated at 4° C overnight with primary anti-PrP antibodies in Superblock. After washing with PBS, cells were labelled with AF488-conjugated anti-mouse IgG (H+L) at a 1:1,000 dilution overnight at 4° C. All images were acquired with the same photomultiplier tube (PMT) gain to account for differences in labelling intensities.

(B-E) Phenotypic differences of PrP^d aggregates detected with distinct anti-PrP mAbs. Chronically prion-infected (upper panels) and uninfected (lower panels) cells were fixed and denatured according to Methods and labelled with anti-PrP antibodies 5B2 (A), 6D11 (B), ICSM18 (C) and 8H4 (D), followed by AF488-conjugated secondary antibodies and DAPI for nuclei. Panels a and b represent magnifications and are denoted in the left panels by dashed insets. All images were acquired with the same photomultiplier tube (PMT) gain to account for antibody-dependent differences in fluorescence intensities.

(F) Serial confocal images of prion-infected S7 cells, double-labelled with anti-focal adhesion kinase (FAK, red spots) and 5B2 (green) at the level of the ECM. In-focus detection of FAK coincides with in-focus detection of PrP^d and is arbitrarily denoted “zero µm” as a focal reference level with an estimated standard deviation of ± 0.2 µm (see “Image acquisition using laser-scanning microscopy in Methods).

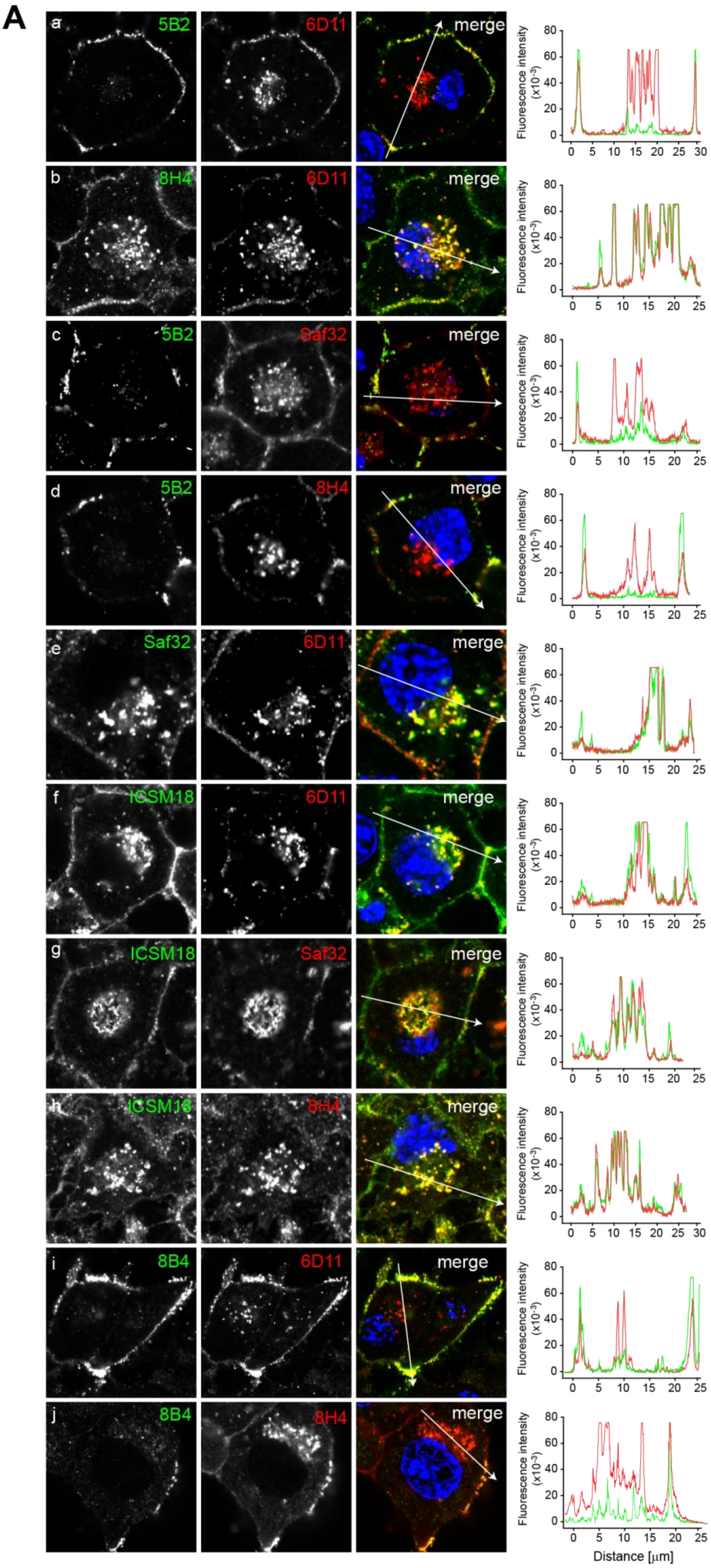
A
a
5B2
6D11
merge
b
8H4
6D11
merge
c
5B2
Saf32
merge
d
5B2
8H4
merge
e
Saf32
6D11
merge
f
ICSM18
6D11
merge
g
ICSM18
Saf32
merge
h
ICSM18
8H4
merge
i
8B4
6D11
merge
j
8B4
8H4
merge
Fluorescence intensity (x10⁻³)
Distance [µm]

Supplementary Fig. 2: Antibodies against the N-terminal region of PrP fail to label perinuclear deposits of PrP^{d}

Aliquots of 5 x 10^{4} persistently prion-infected S7 cells per ml OFCS were plated out into wells of 8-well chamber slides and cultured for 4 days. After fixation and denaturation with 3.5 M GTC as specified in Methods, cells were labelled overnight with the specified primary antibodies. After washing with PBS, cells were labelled overnight with isotype-specific fluorescence-conjugated antibodies (AffiniPure, Jackson ImmunoResearch). Note that all pairwise mAb combinations displayed have different antibody isotypes, i.e. IgG1 (5B2, ICSM18, 8B4), IgG2a (6D11) and IgG2b (8H4, Saf32), while mAbs with the same isotype could not be used for co-labelling. Arrows in images "merge" depict the orientation and placement of fluorescence intensity profiles, shown on the right hand side of image panels.

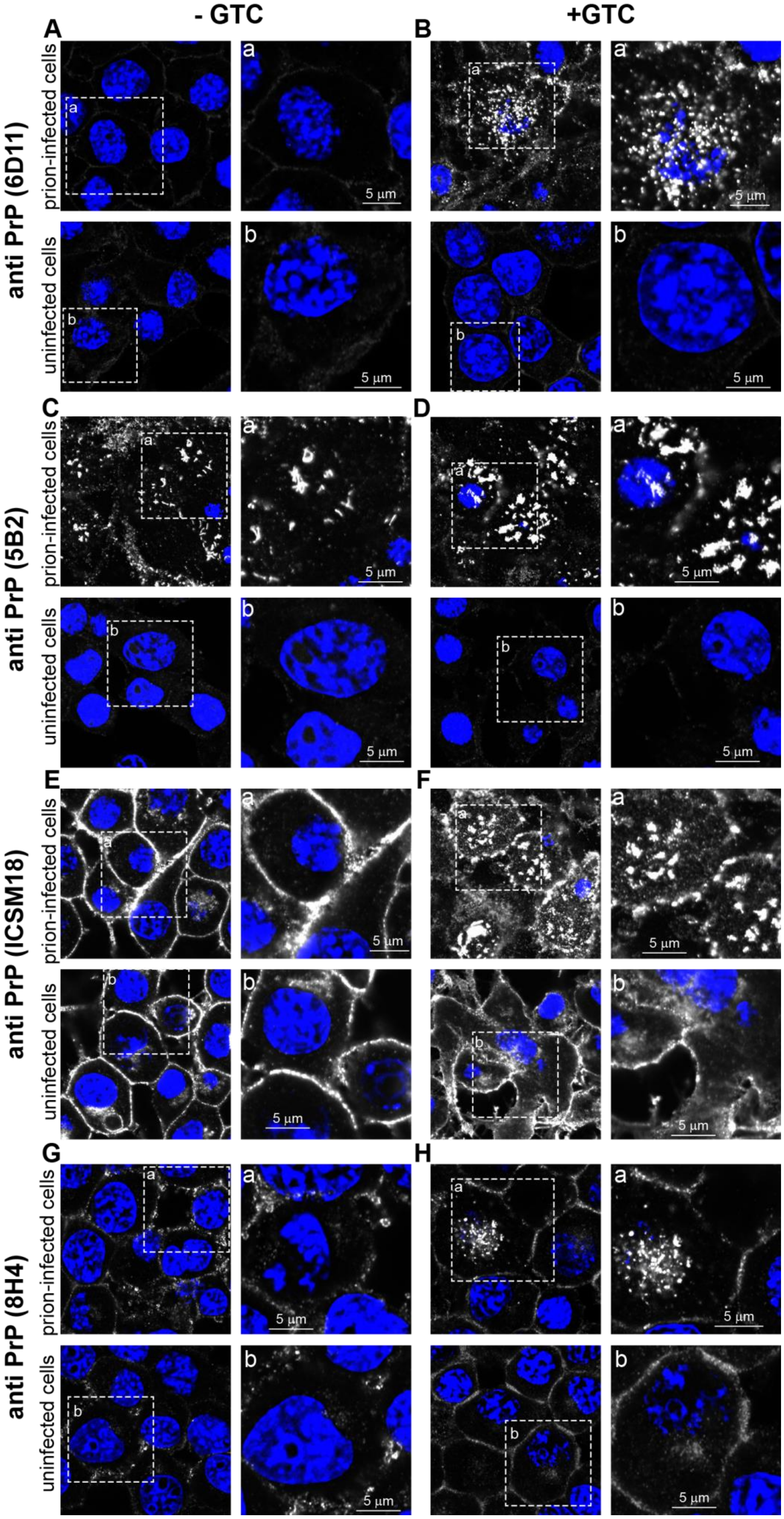

- GTC
+GTC
A
B
C
D
E
F
G
H
anti PrP (6D11)
anti PrP (5B2)
anti PrP (ICSM18)
anti PrP (8H4)
prion-infected cells
uninfected cells
a
b
5 μm

Supplementary Fig. 3: Identification of cryptic epitopes in PrP^d aggregates

(A-H) Confluent layers of prion-infected S7 (top panel) and uninfected S7 (bottom panel) cells were fixed, permeabilised and incubated for 10 minutes with PBS (A, C, E, G) and 3.5 M GTC (B, D, F, H), respectively. Cells were then immunolabelled overnight with primary mAbs 6D11 (A, B), 5B2 (C, D), ICSM 18 (E, F) and 8H4 (G, H), followed by incubation with AF488-conjugated secondary antibody (IgG (H+L), Jackson ImmunoResearch). Hatched insets denote magnified areas which are displayed on the right of the original image.

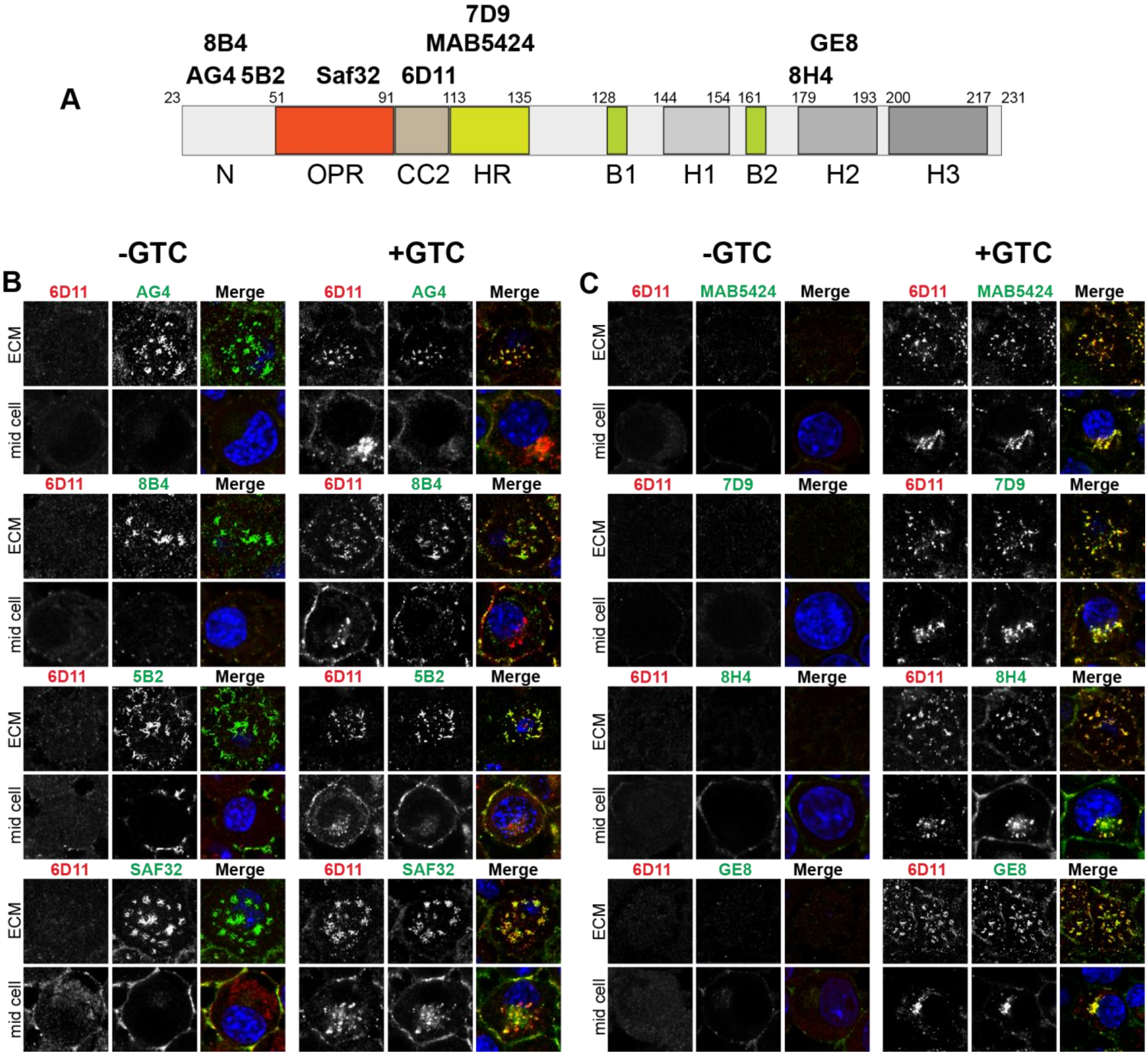

Supplementary Fig. 4: Binding of N-terminal and core anti-PrP mAbs to aggregated PrP^d under native and denaturing conditions

(A) Schematic diagram of anti-PrP mAbs used in this figure and their epitopes on the context of mouse PrP domains, abbreviated as follows: N: N-terminus, OPR: octapeptide repeat region, CC2: charge cluster 2, HR: hydrophobic region, B1: β-strand 1, H1: α-helix 1, B2: β-strand 2, H2: α-helix 2, H3: α-helix 3. MAbs with minor off-target effects were included where detection of aggregated PrP^d showed significantly higher fluorescence than that of PrP^c.
(B, C) Prion-infected S7 cells were fixed with formalin and treated with 3.5 M GTC and PBS, respectively. Cells were then double-labelled with the discriminatory mAb 6D11 as a reference and with any one of the following mAbs: (B) AG4, 8B4, 5B2 and Saf32 and (C) MAB5424, 7D9, 8H4 and GE8. After labelling overnight, cells were washed twice and labelled with isotype-specific secondary antibodies. PrP^d binding of mAbs was imaged at two distinct focal levels, ECM and “mid cell”. At mid cell level, perinuclear sites as well as plasma membranes are clearly visible. The two focal levels thus include all sites where PrP^d deposits were detected, i.e. the plasma membrane, perinuclear sites and the ECM. Representative single cell images are shown.

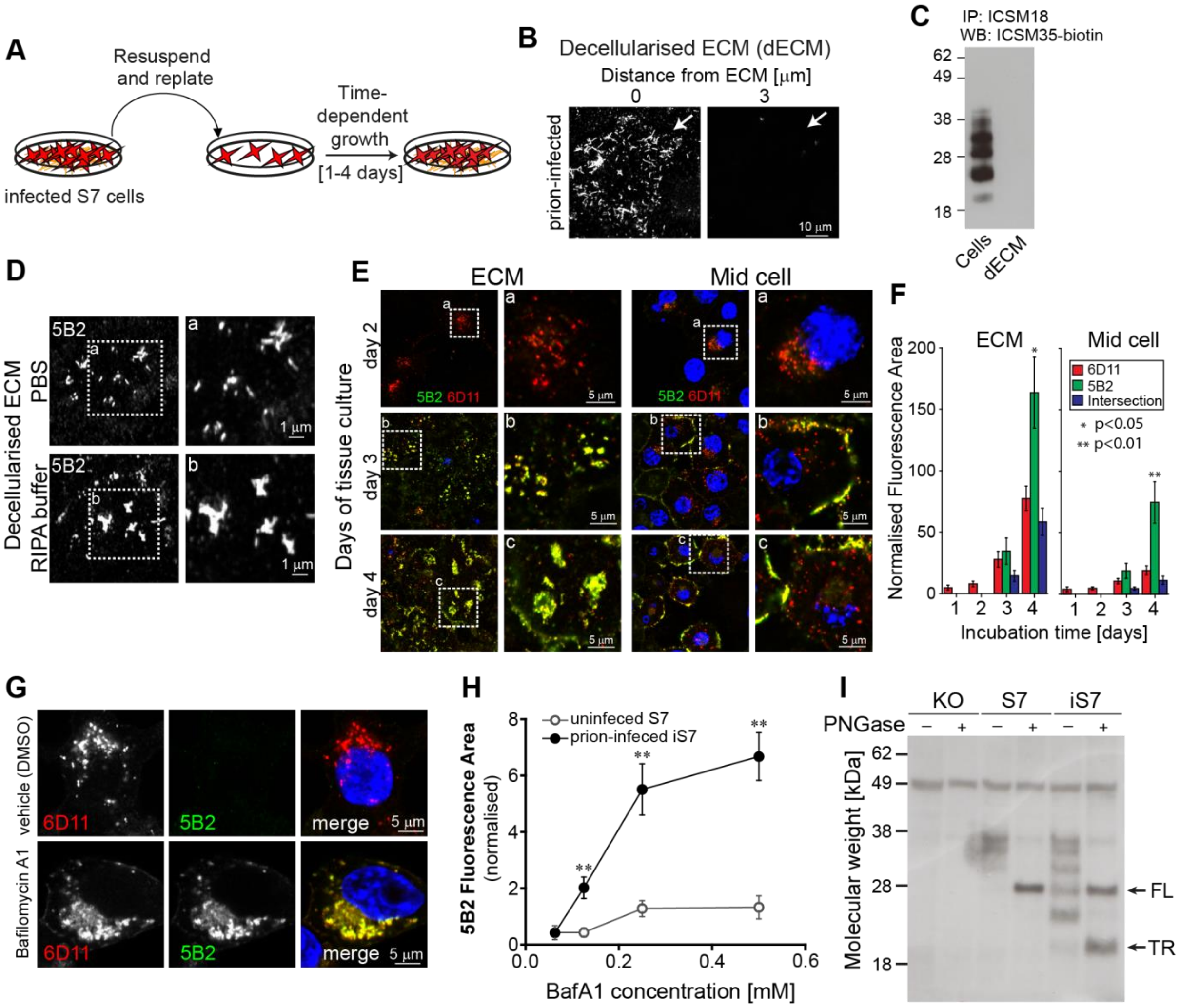

Supplementary Fig. 5: Asynchronous changes in intra- and extracellular PrP^d pools after cell dissociation

(A) As depicted in the schematic, confluent layers of prion-infected S7 cells were split to examine changes in the levels of 6D11- and 5B2-positive PrP^d aggregates upon cell dissociation.

(B) Representative confocal image of a detached prion-infected cell with remaining 5B2-positive fibrils at the ECM. Arrow denotes 5B2 immunopositive fibrils of a detached cell.

(C) Following decellularisation of infected S7 cells, the remaining ECM was lysed with RIPA buffer (dECM) for 15 min. As control, confluent layers of infected S7 cells were lysed in RIPA buffer (cells). Lysates were incubated with ICSM18 and isolated with μMACS Protein G microbeads (Miltenyi). Western blots were labelled with biotinylated ICSM35, followed by avidin/biotin ABC complex (see Methods).

(D) Following cell trituration of infected S7 cells dECM was resuspended in cold RIPA buffer and PBS (control), followed by labelling with 5B2.

(E) Prion-infected S7 cells, labelled with 5B2 and 6D11 at various days post cell dissociation were imaged at two distinct focal planes, ECM and mid cell level; magnified areas are denoted by dashed box.

(F) Quantitative analysis of time-dependent changes in the levels of 5B2-, 6D11-positive and co-labelled fluorescence areas following resuspension and replating of infected cells. One of 3 independent experiments with at least 10 cells per condition analysed is shown. Statistical significance is calculated based on two-tailed p-value using Student's t-test with significance levels denoted.

(G) Representative images of cells, treated with 0.25 µM BafA1 and vehicle, respectively.
(H) Quantitative analysis of 5B2 fluorescence at 2 days after BafA1 treatment of infected and uninfected cells. Statistical analysis of one out of 3 independent experiments is shown with significantly different changes ($p < 0.01$) indicated.
(I) Lysates of confluent Prnp$^{-/-}$ (KO), uninfected (S7) and infected S7 (iS7) cells were prepared and treated in presence and absence of PNGase F as specified in Methods. Lysates were separated on 12% Bis-Tris gels, blotted and labelled with ICSM35, followed by AP-conjugated anti-mouse antibody. Arrows depict full-length (FL) and truncated (TR) PrP. Source data are provided as a Source Data file.

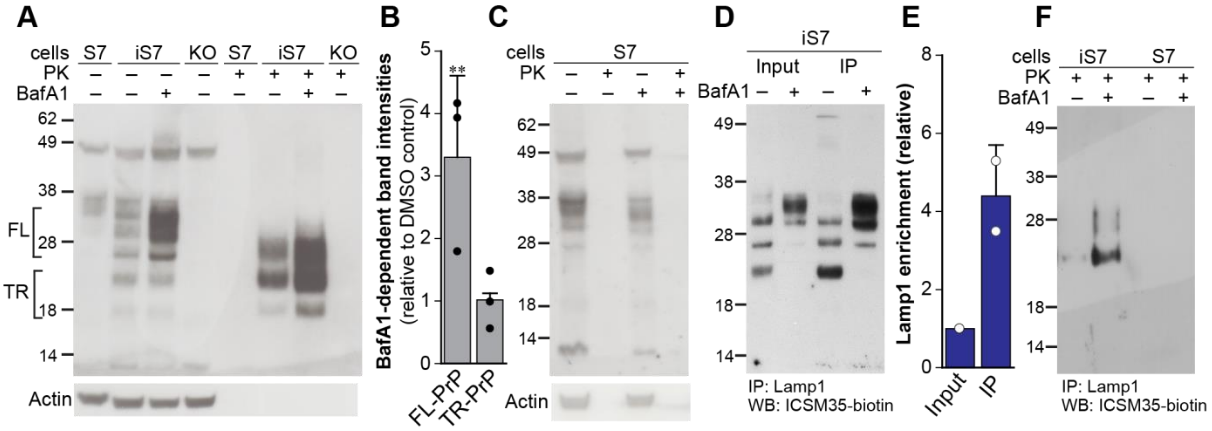

Supplementary Fig. 6: Bafilomycin treatment of prion-infected cells blocks proteolytic processing of FL-PrP^{d} in intracellular sites.

(A) Cells (KO, S7 and iS7) were incubated with 0.5 mM BafA1 or DMSO (0.05 %) for 1 h, washed with complete medium and further incubated for 16 h. Cells were lysed in RIPA buffer and cell lysates treated in absence and presence of 10 µg/ml Proteinase K (PK) for 30 min at 37 °C. Protein digestion was stopped with AEBSF and protein lysates were separated and blotted as described above. Following development of blots with ICSM35, blots were stripped with Restore stripping buffer and reprobed with anti-mouse actin as loading control. Corresponding bands of full-length (FL) and truncated (TR) PrP are denoted by brackets.

(B) Quantitative analysis of relative band intensities after BafA1 treatment, compared to vehicle control in cell lysates without PK digestion. Combined band intensities for FL- and TR-PrP bands as denoted by the bracket in image K were determined. Mean values + Stdv of relative band intensities from three independent experiments are shown. Significant difference ($p<0.001$; Student T-test) is denoted.

(C) Uninfected S7 cells were incubated with 0.5 mM BafA1 or DMSO (0.05 %) for 1 h, washed with complete medium and incubated for an additional 16 h. Cells were further processed as described in A.

(D) Prion-infected S7 cells were incubated with 0.5 mM BafA1 or DMSO essentially as described in A and lysosomes were enriched by immunoprecipitation using anti-Lamp1 antibody ab24170 (Abcam) as specified in Methods. Following immunoblotting, PrP was revealed with biotinylated anti-PrP antibody ICSM35 as described in Methods.

(E) Relative enrichment level of Lamp1 following immunoprecipitation (IP) with anti-Lamp1 antibody ab24170 is depicted. Data of two independent IPs is plotted.

(F) On-column PK digestion of BafA1- or DMSO treated uninfected (S7) and prion-infected (iS7) cells. For technical details, see “Lysosome isolation” in Methods. Levels of PrP^{Sc} were revealed with biotinylated anti-PrP antibody ICSM35. Source data are provided as a Source Data file.

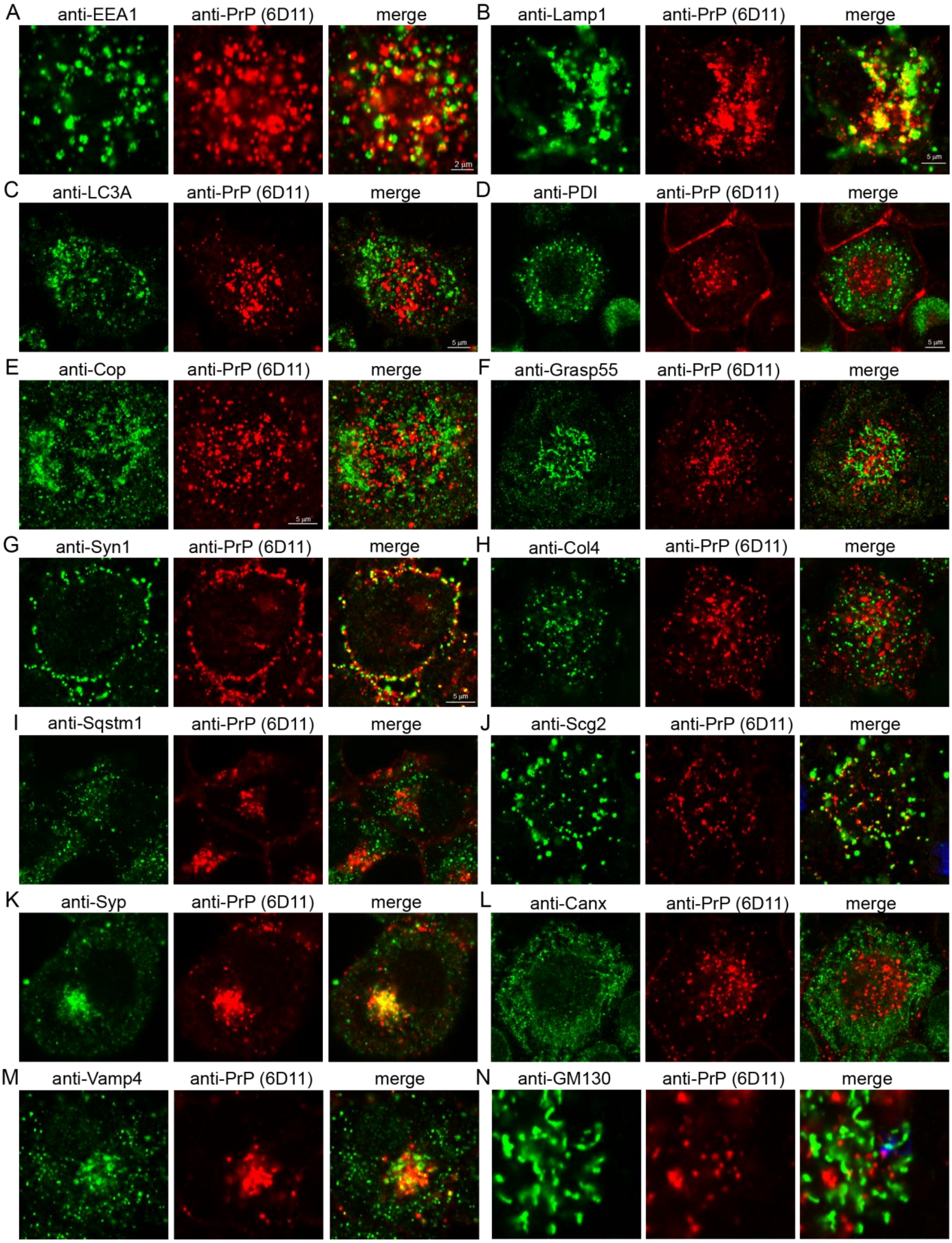

Supplementary Fig. 7: Identification of overlapping distributions of PrP^d with organelle markers to map intracellular trafficking routes of PrP^d

(A-N) Chronically prion-infected cells were fixed and processed according to Methods and labelled with the specified primary antibodies (green label in left image) and 6D11 (red label in centre image). Following washing with PBS highly cross-adsorbed fluorescence-conjugated secondary antibodies (AffiniPure, Jackson ImmunoResearch Laboratories) were incubated overnight at a dilution of 1:1,000 PBS/Superblock (4:1). Representative images

are shown. Corresponding Pearson correlation coefficients are shown in Fig. 4H. Organelle markers used: (A) Eea1: Early endosome antigen 1 (B) Lamp1: Lysosomal associated membrane protein 1 (C) LC3A/ MAP1LC3A: Microtubule Associated Protein 1 Light Chain 3 Alpha (D): Pdi/P4hb: Protein disulphide isomerase (E): β-Cop/Copb1: Coatomer protein complex subunit beta 1 (F): Grasp55/Gorasp2: Golgi reassembly stacking protein (G) Syn1: Synapsin 1 (H) Col4: Collagen 4 (I) Sqstm1: Sequestosome 1 (J): Scg2: Secretogranin 2 (K) Syp: Synaptophysin (L) Canx: Calnexin (M) Vamp4: Vesicle-associated membrane protein 4 (N) Gm130/Golga2: Golgin A2.

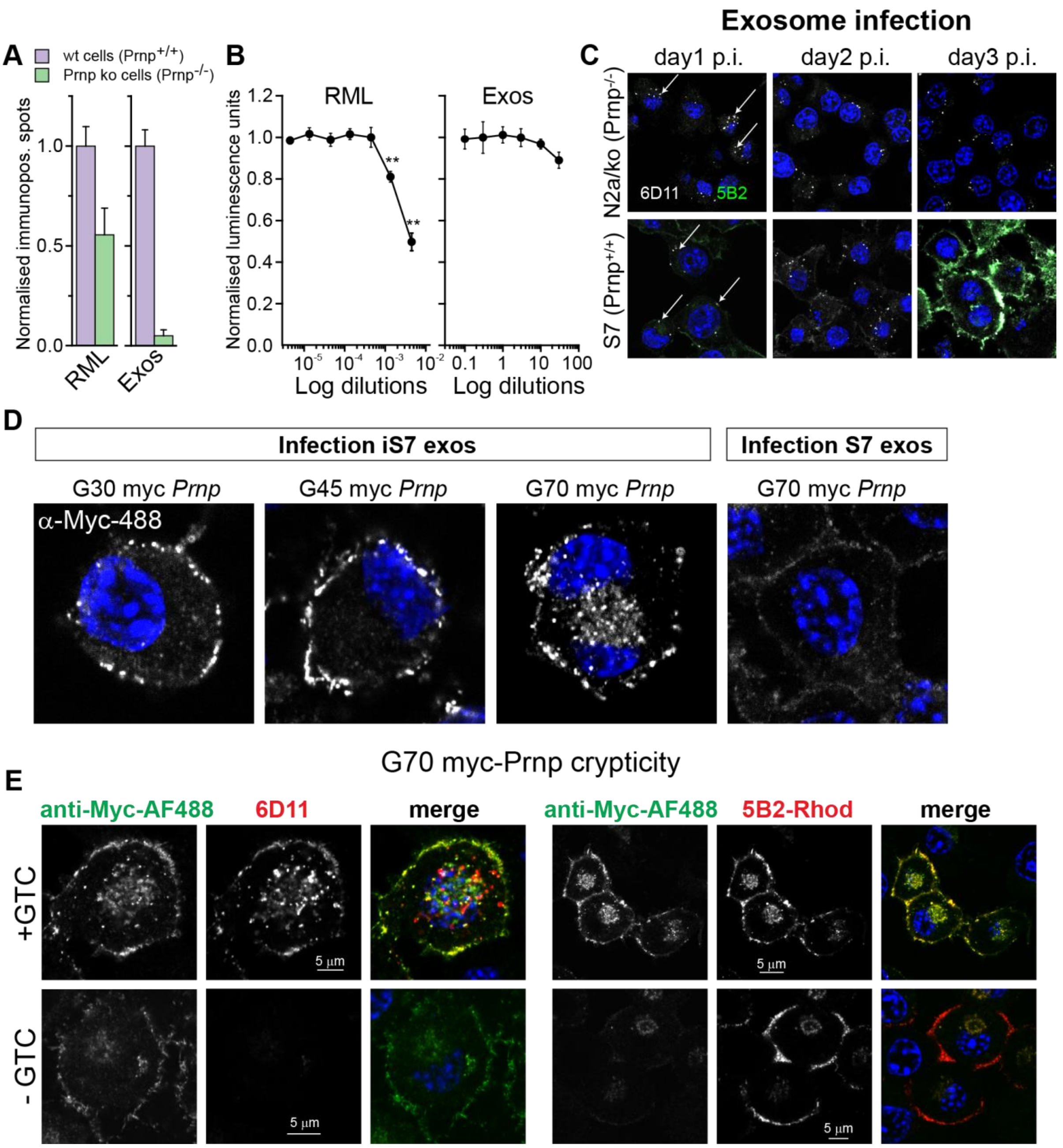

Supplementary Fig. 8: Infection of S7 and G70 *Prnp* expressing cells with prion-infected exosomes

(A) To determine levels of residual inoculum after prion infection, prion-susceptible S7 ($Prnp^{+/+}$) and prion-refractory N2a/ko ($Prnp^{-/-}$) cells were infected with 7.5×10^5 Tissue culture infectious units (TCIU)/ml RML and infectious exosomes, respectively and the number of 6D11-positive spots was determined 3 days after infection by SCA. Data represents average spot counts ± SD, normalised to the corresponding spot numbers of S7 cells. Spots in refractory N2a/ko cells represent inoculum background. One representative experiment with 12 technical repeats per sample is shown.

(B) RML and exosome stock solutions, containing 2.3×10^6 TCIU/ml were serially diluted and S7 cells were infected. After 24h toxicity was determined using CellTiter-Glo luminescent cell viability assay. Data represent normalised average luminescent units ± SD from two independent experiments. Significant changes (** $p<0.001$) were determined using one-way ANOVA.

(C) Prion-susceptible (S7) and -refractory (N2a/ko) cells were incubated with 30 µl exosome concentrate/ml medium, corresponding to an infectious titre of $10^{6.36}$ TCIU/ml. After 1-3 days post infection (p.i.) cells were fixed and labelled with 6D11 and 5B2. Arrows depict 6D11-positive PrP^{d} at the perinuclear region.
(D) Myc PrP-expressing cells, infected with exosomes from persistently infected and uninfected cells, respectively were cultured for 6 passages after infection, fixed and labelled with AF488-conjugated anti-Myc antibody. Cells were imaged at mid-cell level to visualise myc PrP^{d} at perinuclear sites and the plasma membrane.
(F) Persistently infected G70-*Prnp* expressing cells were grown to confluency in 8-well chambered glass slides. Following fixation and acetone treatment, cells were incubated for 10 min with 3.5M GTC or PBS. Cells were thoroughly washed with PBS and labelled with anti-Myc-AF488 and 6D11 (left panel) and with anti-Myc-AF488 and Rho-conjugated 5B2 (right panel). All cells were imaged with the same PMT gains. Source data are provided as a Source Data file.

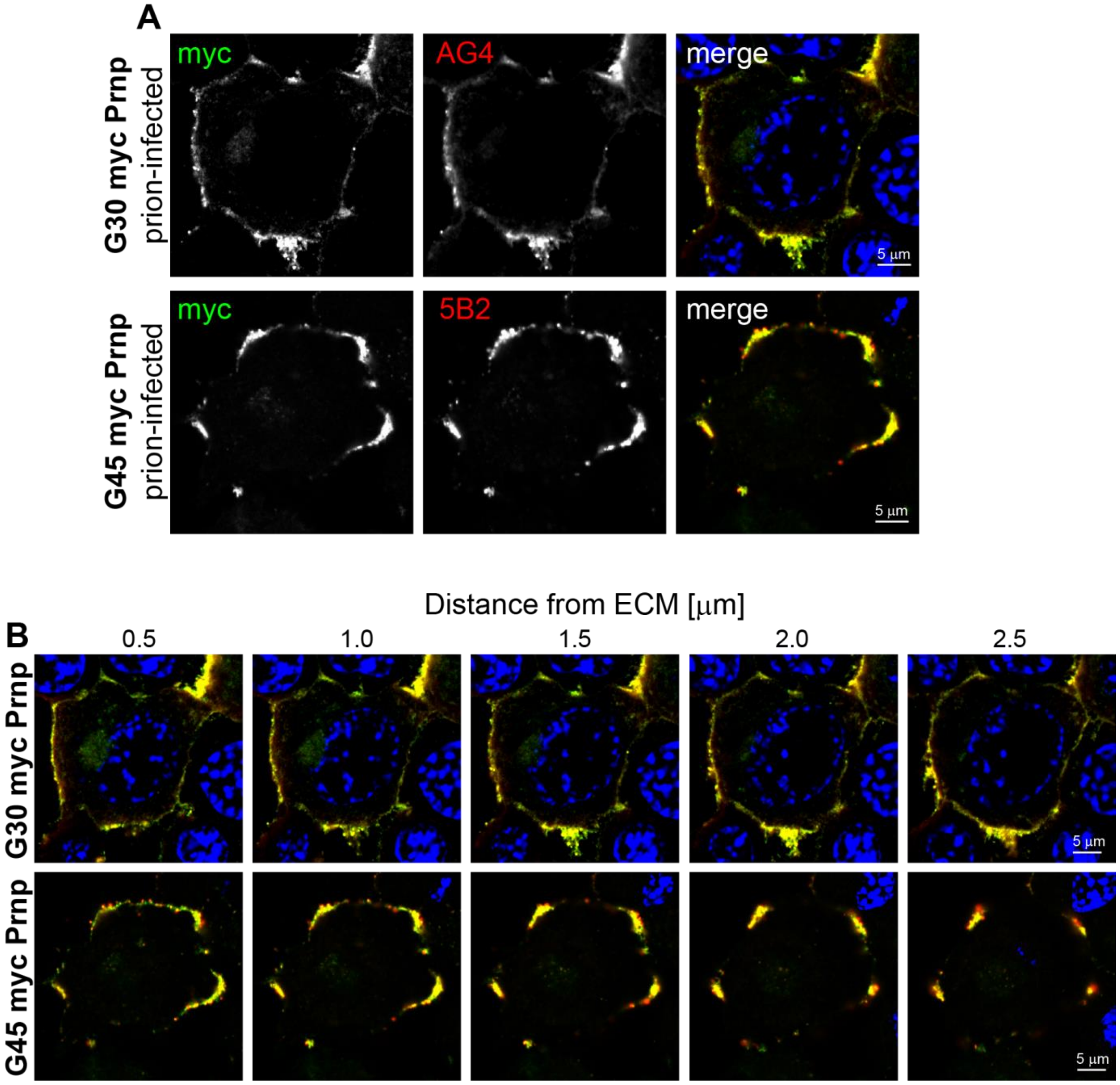

Supplementary Fig. 9: Colabelling of infected G30 and G45 myc *Prnp* expressing cells confirm N-terminal truncation of myc PrP^d.

(A) Formalin-fixed and GTC-denatured persistently infected G30 and G45 myc *Prnp* expressing cells were double labelled with anti-myc and AG4 (32-52) or 5B2 (48-50).

(B) Serial confocal sections of G30 and G45 myc *Prnp* expressing cells, co-labelled with AF488-conjugated anti-myc antibody and anti-PrP mAbs AG4 (top panel) and 5B2 (bottom panel), respectively.

Supplementary Tables

**Name**	**†Mono-specific**	**PrP epitope (§domain)**	**‡Host, Isotype**	***Vendor, Order #**
**5B2**	**+**	48–50[a] (N-term)	M, IgG1	SC, Sc-47730
**6D11**	**+**	93–109[b] (CC2)	M, IgG2a	SC, Sc-58581
**ICSM18**	**+**	146–159[c] (H1)	M, IgG1	MRC, N/A
**8H4**	**+**	175–185[d] (H2)	M, IgG2b	Sigma, P0110
**Saf32**	**+**	59–89[e] (OPR)	M, IgG2b	Cayman, 189720
**8B4**	**+**	37–44[f] (N-term)	M, IgG1	SC, Sc-47729
**AH6**	**+**	N/A	M, IgG2a	SC, Sc-69896
**AG4**	**+/-**	32-52[g] (N-term)	M, IgG2b	TSE RC, RC 059
**7D9**	**+/-**	112-124[g] (HR)	M, IgG1	Abcam, ab14219
**MAB5424**	**+/-**	105-125[b] (CC2, HR)	M, IgG1	Merck, MAB5424
**GE8**	**+/-**	183-191[g] (H2)	M, IgG2b	TSE RC, RC 061
**ab3531**	**-**	90-102[h] (CC2)	R, IgG	Abcam, ab3531
**EP1802Y**	**-**	222–226[l] (C-term)	R, IgG	Novus, NB110-57436
**AB5058**	**-**	79-97[m] (OPR, CC2)	G, IgG	Merck, AB5058
**H-8**	**-**	N/A	M, IgG2a	SC, Sc-393165
**G-12**	**-**	N/A	M, IgG1	SC, Sc-398451
**Pri-917**	**-**	217–226[n] (C-term)	M, IgG1	Spi, AO329
**PrP 27-30**	**-**	N/A	G, IgG	Fitzgerald, 20-PG73

†Monospecific: An anti-PrP antibody is defined monospecific, if no residual signal (off-target binding) is detected in *Prnp*-ablated cells. Antibodies with minor off-target effects are denoted +/-.

§Abbreviation of PrP domains: N-term: N-terminus; OPR: octapeptide repeat region; CC2: charge cluster 2; HR: hydrophobic domain; H1: helix 1; H2: helix 2; C-term: C-terminus.

‡Hosts: M = mouse, R = rabbit, G = goat

*Vendors: SC: Santa Cruz Biotechnology, MRC: MRC Prion Unit, Sigma: Sigma Aldrich, Cayman: Cayman Chemicals, Novus: Novus Biologicals, Spi: Spi Bio, TSC RC: TSE Resources Centre (University of Edinburgh, UK).

N/A: not applicable

[a]Le et al, 2000[1]; [b]Lauren et al., 2009[2]; [c]Antonyuk et al., 2009[3]; [d]Pan et al., 2004[4]; [e] Kang et al., 2013[5]; [f] Zanusso et al., 1998[6]; [g] Silva et al., 2012[7]; [h] Katorcha et al., 2014[8]; [l] Faris et al, 2017[9]; [m] Sauer et al., 1999[10]; [n] Demart et al., 1999[11]

Supplementary Table 1: Validating the target specificity of anti-PrP antibodies using *Prnp* knockout cells. Anti-PrP antibodies were validated following genetic deletion of *Prnp* from N2a cells using CRISPR-Cas9 (see Methods and Supplementary Fig. 1A). To validate antibody specificity, fixed and permeabilised N2a $Prnp^{-/-}$ (ko) and N2a $Prnp^{+/+}$ (wild-type) cells were incubated with anti-PrP overnight. Cells were then washed twice with PBS and incubated with AF488-conjugated secondary antibody. Residual fluorescence in $Prnp^{-/-}$ N2a cells indicates off-target binding, while complete abrogation of fluorescence confirms the mono-specificity of mAbs.

anti-PrP mAb	Cellular Compartment	Mean fluorescence intensity ± SEM [voxels/µm^2]*10^{-5}		T-test	Ratio ∫MFI (infected/ uninfected) ± SEM
		Uninfected	*Infected*		
**6D11**	‡PM	5.9 ± 1.0	82.7 ± 5.6	3.9 x 10^{-4}	14 ± 3
	*Peri	6.2 ± 1.4	160.0 ± 14.9	1.2 x 10^{-7}	27 ± 8
	#ECM	3.9 ± 1.0	114.1 ± 6.6	5.6 x 10^{-6}	29 ± 4
**5B2**	PM	4.9 ± 0.4	65.2 ± 6.1	4.4 x 10^{-13}	13 ± 2
	Peri	3.9 ± 0.5	4.2 ± 7.9	0.8	1 ± 1
	ECM	5.0 ± 0.4	119.0 ± 6.5	3.8 x 10^{-12}	23 ± 2
**Saf32**	PM	22.3 ± 1.0	75.9 ± 4.1	2.4 x 10^{-10}	3 ± 0
	Peri	7.6 ± 0.9	51.9 ± 5.0	1.3 x 10^{-3}	7 ± 2
	ECM	10.0 ± 3.7	117.5 ± 3.4	2.3 x 10^{-10}	12 ± 1
**ICSM18**	PM	27.0 ± 1.7	47.9 ± 4.1	0.2	2 ± 1
	Peri	13.6 ± 6.0	36.8 ± 3.4	6.9 x 10^{-3}	3 ± 1
	ECM	11.8 ± 1.7	99.9 ± 7.4	8.0 x 10^{-3}	8 ± 1
**8H4**	PM	29.4 ± 2.1	60.4 ± 3.7	1.5 x 10^{-8}	2 ± 0
	Peri	5.7 ± 1.0	125.3 ± 17.7	3.6 x 10^{-4}	22 ± 5
	ECM	12.7 ± 1.3	66.9 ± 2.3	1.5 x 10^{-8}	5 ± 0

‡ PM: Plasma membrane; *Peri: Perinuclear region; # ECM: Extracellular matrix; ∫ MFI: Mean fluorescence intensity

Supplementary Table 2: Voxel-based fluorescence intensities of anti-PrP antibodies for cellular compartments where PrP^d deposits. Uninfected and prion-infected S7 cells were grown in OFCS for 4 days. Cells were then fixed with formalin (3.7%) for 12 min, followed by a 1 min and 10 min treatment with acetone and GTC (3.5M), respectively. After thorough washing with PBS, cells were labelled for 12 h with anti-PrP antibodies 6D11, 5B2, Saf32, ICSM18 and 8H4. After washing with PBS, cells were labelled for 12 h with a Jackson Affinipure anti-M IgG (H+L) AF488 antibody and DAPI. Fluorescence intensities were set for the antibody with highest fluorescence intensities (6D11 in infected cells), using range indicator and kept the same for all other antibodies. Statistical significance was assessed by Student's T-test.

Inhibitor	Concentration	Cytotoxicity [% of control]	Toxicity above threshold
**Bafilomycin A1**	24 nM	21.0	Yes
	12 nM	8.8	No
	6 nM	4.4	No
**NH_4Cl**	32 mM	25.0	Yes
	16 mM	1.4	No
	8 mM	0.8	No
**Dynasore**	40 µM	17.5	Yes
	20 µM	0.0	No
	10 µM	0.0	No
**Dynole**	5 µM	10.8	Yes
	2.5 µM	0.0	No
	1.25 µM	0.0	No
**Pitstop 2**	10 µM	12.5	Yes
	5 µM	7.5	No
	2.5 µM	3.2	No
**Cytochalasin D**	50 nM	0.8	No
	5 nM	2.0	No
	0.5 nM	7.1	No
**Amiloride**	40 µM	5.4	No
	20 µM	0.0	No
	10 µM	0.0	No
**Vacuolin**	5 µM	1.9	No
	2.5 µM	0.0	No
	1.25 µM	0.0	No
**EGA**	10 µM	2.3	No
	5 µM	1.6	No
	2.5 µM	3.5	No
**Golgicide**	20 µM	0.0	No
	10 µM	0.0	No
	5 µM	0.0	No
**mβCD**	4 mM	51.2	Yes
	2 mM	3.2	No
	1 mM	0.0	No
**Retro-2**	10 µM	8.0	No
	5 µM	2.3	No
	2.5 µM	0.2	No

Supplementary Table 3: Toxic threshold levels of small molecule inhibitors

The cytotoxicity of small molecule inhibitors was assessed by measuring ATP levels by Luminescent Cell Viability Assay (Cell Titre Glo, Promega). Cells were plated out at a concentration of 5 x 10^4 cells per ml OFCS. Serially diluted inhibitors were added 16 h after

plating and the experiment was terminated after 3 days. To take experimental variation into account, cytotoxic effects were scored positive, when a threshold of 10% was exceeded, when compared to mock controls.

Inhibitor	Concentration	Fold Change	T-test
Bafilomycin A1	100 nM	0.96	5.5×10^{-1}
	10 nM	0.90	3.6×10^{-1}
	1 nM	0.99	8.6×10^{-1}
	100 pM	1.00	9.1×10^{-1}
NH_4Cl	16 mM	0.99	8.8×10^{-1}
	8 mM	0.86	1.2×10^{-1}
	4 mM	0.93	0.9×10^{-1}
	2 mM	1.05	5.0×10^{-1}
Dynasore	5 µM	0.12	1.8×10^{-17}
	2.5 µM	0.11	8.0×10^{-17}
	1.25 µM	0.12	1.2×10^{-16}
	0.6 µM	0.16	5.9×10^{-16}
Dynole	2.5 µM	0.03	7.9×10^{-17}
	1.25 µM	0.26	2.0×10^{-12}
	0.6 µM	0.63	6.2×10^{-5}
	0.3 µM	0.92	3.5×10^{-1}
Pitstop 2	2.5 µM	1.24	1.0×10^{-1}
	1.25 µM	1.22	8.0×10^{-2}
	0.6 µM	1.11	3.2×10^{-1}
	0.3 µM	1.29	7.0×10^{-2}
Cytochalasin D	40 nM	0.60	8.0×10^{-11}
	20 nM	0.71	1.6×10^{-6}
	10 nM	0.77	1.6×10^{-6}
	5 nM	0.84	4.3×10^{-4}
Amiloride	20 µM	0.87	8.4×10^{-5}
	10 µM	0.90	4.7×10^{-4}
	5 µM	0.91	8.4×10^{-4}
	2.5 µM	0.93	6.3×10^{-2}
Vacuolin	5 µM	0.16	3.2×10^{-11}
	2.5 µM	0.19	8.4×10^{-11}
	1.25 µM	0.51	2.4×10^{-5}
	0.625 µM	0.74	1.2×10^{-2}
EGA	5 µM	0.37	3.6×10^{-14}
	2.5 µM	0.63	4.1×10^{-3}
	1.25 µM	0.93	4.4×10^{-1}
	0.625 µM	1.26	9.7×10^{-2}
Golgicide	5 µM	0.33	8.2×10^{-4}
	2.5 µM	0.76	1.5×10^{-1}
	1.25 µM	0.95	7.8×10^{-1}
	0.625 µM	1.21	3.7×10^{-1}
mβCD	2 mM	0.14	1.8×10^{-18}
	1 mM	0.40	8.2×10^{-11}
	0.8 mM	0.64	2.7×10^{-7}
	0.6 mM	0.80	6.2×10^{-5}
	0.4 mM	0.96	5.5×10^{-1}

Supplementary Table 4: Effect of small inhibitory molecules on the cellular prion steady state levels

Effects of small molecule inhibitors on the prion steady state levels were determined by SCA. Stock solutions of inhibitors were prepared in DMSO with the exception of NH_4Cl. To determine effects of small molecule inhibitors on prion steady state levels, 300 µl aliquots of chronically infected S7 cells were plated into wells of 96-well plates at a concentration of 5 x 10^4 cells per ml OFCS. After 16 hours, cells were incubated with serially diluted inhibitors. After 3 days, cells were resuspended, diluted 1:10 in PBS and 100 µl of the cell suspension was transferred onto ELISPOT plates (Cat#. MSIPN4550, Merck Millipore Ltd, Tullagreen, Ireland) and prion titres were determined. Data represent fold change values, compared to mock (DMSO) treatment of at least three independent experiments.

Control gene	Gene	Gene name	NCBI gene ID	Knockdown [%]
ActB	Prnp	Prion protein	19122	86.0 ± 3.6
	Clta	Clathrin, light polypeptide	12757	97.5 ± 0.9
	Cdc42	cell division cycle 42	12540	96.6 ± 2.1
	Dnm1	Dynamin 1	13429	95.4 ± 1.3
	Dnm2	Dynamin 2	13430	90.0 ± 0.1
	Rhoa	Ras homolog family member A	11848	95.3 ± 4.7
	†Arf1	ADP-ribosylation factor 1	11840	89.3 ± 5.1
	†Arf6	ADP-ribosylation factor 6	11845	81.7 ± 9.9
	Cav1	Caveolin 1	12389	N/A
Gapdh	Prnp	Prion protein	19122	84.2 ± 4.6
	Clta	Clathrin, light polypeptide	12757	97.8 ± 0.2
	Cdc42	cell division cycle 42	12540	96.5 ± 2.7
	Dnm1	Dynamin 1	13429	95.01 ± 0.9
	Dnm2	Dynamin 2	13430	89.4 ± 0.9
	Rhoa	Ras homolog family member A	11848	94.9 ± 2.3
	†Arf1	ADP-ribosylation factor 1	11840	89.0 ± 5.0
	†Arf6	ADP-ribosylation factor 6	11845	80.0 ± 11.4
	Cav1	Caveolin 1	12389	N/A

N/A: not applicable: gene not expressed in S7 cells

† For Arf1 and Arf2, siRNA pools are custom-designed against the 3'-UTR regions of said genes, while all other siRNA pools are designed against gene coding regions.

Supplementary Table 5: Efficacy of gene silencing by real-time quantitative PCR

Levels of gene expression following transcriptional silencing with siRNA pools (siTools Biotech GmbH, Planegg, Germany) were determined by real-time quantitative PCR (RT-qPCR). Complementary DNA (cDNA) was synthesised with QuantiTect reverse transcription kit (Qiagen, Manchester, UK) using 500 ng of total RNA. All RT-qPCR reactions were carried out using a QuantStudio 12K Flex (Applied Biosystems, Cheshire, UK) with the following cycling parameters: 50°C, 2 mins; 94°C, 15 min; 40 cycles at 94°C, 15 sec; 60°C, 1 min. Samples were set up in triplicates in 10 µl reactions containing QuantiTect SYBR Green (Qiagen), 1x QuantiTect customised Primer Assay (lyophilized in TE, pH 8.0), 25 ng of cDNA made to a final volume with ddH_20. Gapdh and Actb were used as endogenous controls for normalising target gene expression levels. Data acquisition of the fluorescent signal was performed at the end of the run to assess the expression of mRNA by evaluating threshold cycle (CT) values. Double delta CT calculations were measured as logarithm and then converted to fold change after untreated control levels were subtracted from target levels.

**Inoculum**	**Time after infection**	**Cellular distribution of *de novo* myc PrP [% of positive cells]**		
		†Peri	***#PM***	***Peri & PM***
**RML**	2 min	45.0 ± 4.4	40.3 ± 8.6	9.0 ± 8.7
	15 min	14.0 ± 13.9	62.7 ± 28.9	19.0 ± 22.0
**Exosomes**	2 min	55.0 ± 7.2	29.3 ± 16.9	23.3 ± 13.5
	15 min	40.0 ± 5.2	36.7 ± 15.8	25.3 ± 6.8

†Peri: perinuclear; #PM: plasma membrane

Supplementary Table 6: Identification of the cellular sites of *de novo* PrP conversion

The cellular distribution of *de novo* myc PrP in myc PrP/6D11 double-positive G70 PrP-expressing cells at 2 min and 15 min after infection with RML and exosomes was scored by three investigators. Data represents the cellular locations of *de novo* PrP in percent of the total positive cells scored. At least 50 myc PrP^d positive cells were analysed per condition.

Supplementary References

1. Li RL*, et al.* Identification of an epitope in the C terminus of normal prion protein whose expression is modulated by binding events in the N terminus. *Journal of Molecular Biology* **301**, 567-573 (2000).

2. Lauren J, Gimbel DA, Nygaard HB, Gilbert JW, Strittmatter SM. Cellular prion protein mediates impairment of synaptic plasticity by amyloid-beta oligomers. *Nature* **457**, 1128-1132 (2009).

3. Antonyuk SV*, et al.* Crystal structure of human prion protein bound to a therapeutic antibody. *Proceedings of the National Academy of Sciences of the United States of America* **106**, 2554-2558 (2009).

4. Pan T, Li R, Kang SC, Wong BS, Wisniewski T, Sy MS. Epitope scanning reveals gain and loss of strain specific antibody binding epitopes associated with the conversion of normal cellular prion to scrapie prion. *J Neurochem* **90**, 1205-1217 (2004).

5. Kang M, Yeon KS, An SS, Ran JY. Characterizing affinity epitopes between prion protein and beta-amyloid using an epitope mapping immunoassay. *Exp Mol Med* **45**, e34 (2013).

6. Zanusso G*, et al.* Prion protein expression in different species: analysis with a panel of new mAbs. *Proc Natl Acad Sci U S A* **95**, 8812-8816 (1998).

7. Silva CJ. Using small molecule reagents to selectively modify epitopes based on their conformation. *Prion* **6**, 163-173 (2012).

8. Katorcha E, Makarava N, Savtchenko R, Azzo D, Baskakov IV. Sialylation of Prion Protein Controls the Rate of Prion Amplification, the Cross-Species Barrier, the Ratio of PrPSc Glycoform and Prion Infectivity. *PLoS Pathog* **10**, e1004366 (2014).

9. Faris R*, et al.* Cellular prion protein is present in mitochondria of healthy mice. *Sci Rep* **7**, 41556 (2017).

10. Sauer H, Dagdanova A, Hescheler J, Wartenberg M. Redox-regulation of intrinsic prion expression in multicellular prostate tumor spheroids. *Free Radical Biology and Medicine* **27**, 1276-1283 (1999).

11. Demart S*, et al.* New insight into abnormal prion protein using monoclonal antibodies. *Biochem Biophys Res Commun* **265**, 652-657 (1999).
