# Supplementary material for: Prion protein conversion at two distinct cellular sites precedes fibrillisation
Source: Nat Commun. 2023 Dec 15;14:8354. doi: 10.1038/s41467-023-43961-1 (PMC10724300; doi:10.1038/s41467-023-43961-1)
Supplement: Supplementary file 3 — Reporting Summary [file 41467_2023_43961_MOESM3_ESM.pdf]

nature portfolio

Corresponding author(s): Dr Peter-Christian Kloehn

Last updated by author(s): Nov 17, 2023

Reporting Summary

Nature Portfolio wishes to improve the reproducibility of the work that we publish. This form provides structure for consistency and transparency in reporting. For further information on Nature Portfolio policies, see our Editorial Policies and the Editorial Policy Checklist.

Statistics

For all statistical analyses, confirm that the following items are present in the figure legend, table legend, main text, or Methods section.

n/a	Confirmed	
☐	☒	The exact sample size (*n*) for each experimental group/condition, given as a discrete number and unit of measurement
☐	☒	A statement on whether measurements were taken from distinct samples or whether the same sample was measured repeatedly
☐	☒	The statistical test(s) used AND whether they are one- or two-sided<br>*Only common tests should be described solely by name; describe more complex techniques in the Methods section.*
☒	☐	A description of all covariates tested
☐	☒	A description of any assumptions or corrections, such as tests of normality and adjustment for multiple comparisons
☐	☒	A full description of the statistical parameters including central tendency (e.g. means) or other basic estimates (e.g. regression coefficient) AND variation (e.g. standard deviation) or associated estimates of uncertainty (e.g. confidence intervals)
☐	☒	For null hypothesis testing, the test statistic (e.g. F, t, r) with confidence intervals, effect sizes, degrees of freedom and P value noted<br>*Give P values as exact values whenever suitable.*
☒	☐	For Bayesian analysis, information on the choice of priors and Markov chain Monte Carlo settings
☒	☐	For hierarchical and complex designs, identification of the appropriate level for tests and full reporting of outcomes
☐	☒	Estimates of effect sizes (e.g. Cohen's d, Pearson's r), indicating how they were calculated

Our web collection on statistics for biologists contains articles on many of the points above.

Software and code

Policy information about availability of computer code

Data collection	All images were acquired on Zeiss LSM 710 and Zeiss ELYRA PS.1 microscopes; Zeiss image acquisition software Zen with super-resolution PALM/STORM module (Zeiss) was used; for real-time qPCR data, QuantStudio 12K Flex software (Applied Biosystems) was used; Bioreader 5000-Eß system software (BioSys, Karben, Germany) was used for spot detection on ELISPOT; cell counts were acquired with Z series Coulter Counter software (Beckman Coulter). Scrapie cell assays were done fully automated on a Biomek FX liquid handling robot.
Data analysis	Statistical analyses were conducted using SPSS [Version 26], Graphpad Instat, version 3.10 and Microsoft Excel 2010; Images were analyzed using analysis software Volocity 5.3 (Perkin Elmer) and open source software ImageJ; confocal images were analyzed with Zen black (Zeiss).

For manuscripts utilizing custom algorithms or software that are central to the research but not yet described in published literature, software must be made available to editors and reviewers. We strongly encourage code deposition in a community repository (e.g. GitHub). See the Nature Portfolio guidelines for submitting code & software for further information.

Data

Policy information about availability of data

All manuscripts must include a data availability statement. This statement should provide the following information, where applicable:

- Accession codes, unique identifiers, or web links for publicly available datasets
- A description of any restrictions on data availability
- For clinical datasets or third party data, please ensure that the statement adheres to our policy

Data supporting the findings of this study are presented within the article and supplementary information and are available from the corresponding author upon

request. The data underlying Figs. 1G, 2B, 3D, 3FG, 4G, 4J-K, 5A-C, 5E, 5G-J, 6B, 7A-C and Supplementary Figs. 5C, 5F, 5H-O, 7A-B are provided in the Source Data file. The composition of siPools and primer sequences of qPCR assay is proprietary information by siTOOLs Biotech GmbH and Qiagen, respectively. Source data are provided with this paper.

Human research participants

Policy information about studies involving human research participants and Sex and Gender in Research.

Reporting on sex and gender	N/A
Population characteristics	N/A
Recruitment	N/A
Ethics oversight	N/A

Note that full information on the approval of the study protocol must also be provided in the manuscript.

Field-specific reporting

Please select the one below that is the best fit for your research. If you are not sure, read the appropriate sections before making your selection.

- [x] Life sciences
- [ ] Behavioural & social sciences
- [ ] Ecological, evolutionary & environmental sciences

For a reference copy of the document with all sections, see nature.com/documents/nr-reporting-summary-flat.pdf

Life sciences study design

All studies must disclose on these points even when the disclosure is negative.

Sample size	Sample sizes for all experiments are reported in figure legends or Methods. While sample size calculations were not conducted, samples sizes were adequate based on reported effect levels, consistencies of measured differences between experimental groups and associated statistical significance levels. Prior expertise in applied methodologies used have further provided guiding information on sample sizes. Relevant publications: Bhamra et al., 2023 (PMID: 36535427), Schmidt et al., 2015 (PMID: 26631378), Marbiah et al., 2014 (PMID: 24843046), Philiastides et al., 2019 (PMID: 31546723).
Data exclusions	No data were excluded where assays performed within predefined sensitivity values for internal standards and controls. Data were excluded, if and when assays (Scrapie cell assay, transcriptional silencing, toxicity assays) failed technically. As a default experimental design, internal controls and standards were included in assays, such as Prnp knockdown in gene silencing experiments (Fig 5E), prion-infected brain homogenates with known titeres in Scrapie cell assay (Fig 5G-I). Predefined cutoff values: Viability assay (CellTiter-Glo luminescence assay): assays exceeding 10% toxicity threshold; Transcriptional silencing: curing of prion-infected cells within 3-day period following Prnp knockdown less than 60% reduction; Scrapie cell assay: Infection of cells with a 1:1,000 dilution of the mouse prion strain RML (titer: 10exp 7.4 LD50 units/ml) yielding spot numbers below 1,000/well at cell passage three.
Replication	Each experiment was repeated at least three times to ensure reproducibility. All findings were reproducible within the reported experimental paradigms. Additional measures were taken to confirm outcomes: For perturbation studies, pharmacological inhibitors were used in addition to gene knockdown for the same targets (Fig. 5A and E). For the frequency of infection, correlative imaging and prion titer output methodology were used (Fig. 5F-G and Fig. 6E).
Randomization	Selection or sample biases are not deemed relevant in experimental in vitro studies using immortalized cell lines and primary neuronal cultures. Randomization was therefore omitted.
Blinding	Where deemed critical, investigators were blinded to group allocations. Fig. 6D which documents the number of PrP conversion sites is based on blinding of investigators to group allocations. All other data were acquired and analyzed in a non-blinded fashion as they either involved automated acquisition protocols (Scrapie cell assay, tile scans for confocal imaging, batch analysis by Volocity, automated spot detection on the Bioreader 5000-Eß system) or were based on clearly quantifiable endpoints thus excluding subject judgement.

Reporting for specific materials, systems and methods

We require information from authors about some types of materials, experimental systems and methods used in many studies. Here, indicate whether each material, system or method listed is relevant to your study. If you are not sure if a list item applies to your research, read the appropriate section before selecting a response.

Materials & experimental systems

n/a	Involved in the study
☐	☒ Antibodies
☐	☒ Eukaryotic cell lines
☒	☐ Palaeontology and archaeology
☐	☒ Animals and other organisms
☒	☐ Clinical data
☒	☐ Dual use research of concern

Methods

n/a	Involved in the study
☒	☐ ChIP-seq
☒	☐ Flow cytometry
☒	☐ MRI-based neuroimaging

Antibodies

Antibodies used

The antibodies used in this study were summarised as follows (clone, company, catalogue number). Unless otherwise stated, antibodies were used at a 1:1,000 dilution: Mouse monoclonal anti-PrP (8H4, Sigma, P0110), rat monoclonal Lamp1 (1D4B, Santa Cruz Biotechnology (SCB), sc-19992), mouse monoclonal anti-PrP (5B2, SCB, sc-47730; used at a 1:500 dilution), mouse monoclonal anti-PrP (AH6, SCB, sc-69896), mouse monoclonal anti-PrP (8B4, SCB, sc-47729), mouse monoclonal anti-Syp (7.2, Synaptic Systems, 101 011), rabbit polyclonal anti-Vamp4 (Synaptic Systems, 136 002), rabbit polyclonal anti-Chga (Synaptic Systems, 259 003), rabbit polyclonal anti-phospho FAK (Tyr925, Cell Signaling Technologies, 3284), rabbit polyclonal anti-beta Cop (Thermo Fisher Scientific, PA1-061), rabbit polyclonal anti-Scg2 (Abcam, ab12241), mouse monoclonal anti-PrP (7D9, Abcam, ab14219), rabbit polyclonal anti Col4 (AbD Serotech, 2150-1470), rat monoclonal anti-CD29 (9EG7, BD Biosciences, 553715), mouse monoclonal anti-GM130 (35, BD Biosciences, 610822), mouse monoclonal anti-PrP (6D11, BioLegend, 808002), mouse monoclonal anti-PrP (Saf32, Cayman, 189720), rabbit monoclonal anti-PDI (C81H6, Cell Signaling Technology, 3501S), rabbit monoclonal anti-Eea1 (C45B10, Cell Signaling Technology, 3288), rabbit polyclonal anti-Lc3a (Cell Signaling Technology, 4599), rabbit polyclonal anti-GFAP (DAKO, Z0334, used at a 1:10,000 dilution), Mouse monoclonal anti-PrP (3F4, Merck, MAB1562), mouse monoclonal anti-PrP (MAB5424, Merck, MAB5424), mouse monoclonal anti-myc, AF 488-conjugated (9E10, Merck, 16-308), rabbit polyclonal anti-Syp1 (Millipore, AB1543), mouse monoclonal anti-Snap25 (SP14, Millipore, MAB331), rabbit polyclonal anti-Gorasp2 (Grasp55) (Proteintech, 10598-1-AP), mouse monoclonal anti-PrP (AG4, TSE Resources Centre, TSE RC, RC 059), mouse monoclonal anti-PrP (GE8, TSE Resources Centre, TSE RC, RC 061), ICSM18, ICSM35 and ICSM35-B (UCL Institute of Prion Diseases), chicken polyclonal anti-Map2 (Abcam, ab5392).

Validation

To validate the target specificity of all anti-PrP antibodies used in this study (5B2, 8B4, AG4, Saf32, 6D11, MAB5424, 7D9, ICSM18, 8H4, GE8, G-12, Ab3531, H-8, EP1802Y, Pri-917, Prp-27-30), we generated Prnp-knockout cells by CRISPR-Cas9 as described in Materials and Methods. Validation data of anti-PrP antibodies is reported in Supplementary Figure S1A and Table S1. The method for generating Prnp-kockdown cells is documented in Materials and Methods.

Commercial antibody validation data are very scarce. In our strategy for sourcing validated binders (antibodies), the first point of reference is CiteAb (www.citeab.com), a curated and awarded antibody search site that lists all past publications for commercial antibodies. For any given antibody clone, we thus researched the cited published articles for antibody validation data. Evidence-based data on antibody validation was thus the primary route for sourcing antibodies in this study. A principal metric on the reliability of antibodies at CiteAb is the number of citations in scientific journals. Specified below is the number of references for antibodies used in this study.

Lamp1 (1D4B, Santa Cruz Biotechnology (SCB), sc-19992): 187 citations (see CiteAb); FAK (pY397, Thermo Fisher Scientific, 44-624G): 209 citations; Cop (Thermo Fisher Scientific, PA1-061): 67 citations; Scg2 (Abcam, ab12241): 10 citations; Col4 (AbD Serotech, 2150-1470): 128 citations; CD29 (9EG7, BD Biosciences, 553715): 196 citations; GM130 (35, BD Biosciences, 610822): 683 citations; PDI (C81H6, Cell Signaling Technology, 3501S): 158 citations; GFAP (DAKO, Z0334): 2621 citations; Myc (9E10, Merck, 16-308): 7170 citations; Snap25 (SP14, Millipore, MAB331): 38 citations; Gorasp2 (Grasp55) (Proteintech, 10598-1-AP): 48 citations.

The distributor Synaptic Systems provides antibodies that have been validated in cell lines by gene knockdown (kd) of the relevant target. Knockdown-validated antibodies were sourced from Synaptic Systems for this study.

Eukaryotic cell lines

Policy information about cell lines and Sex and Gender in Research

Cell line source(s)

Neuro2a (N2a) cell line and derivatives. N2a cells (male donor) were sourced from ATCC (CCL-131). Prion-susceptible subclones of N2a cells were derived as described in Kloehn et al., PNAS 2003 Sep 30;100(20):11666-71 (doi: 10.1073/pnas.1834432100) and Marbiah et al., EMBO J 2014 Jul 17;33(14):1527-47 (doi: 10.15252/embj.201387150).

Primary cortico-hippocampal cultures were prepared from embryonic e17 FVB mouse brains from female mice.

Authentication

N2a is a broadly available commercial cell line that was purchased from ATCC. Prion-susceptible sublines thereof were generated by subcloning (Kloehn et al., PNAS 2003 Sep 30;100(20):11666-71; doi: 10.1073/pnas.1834432100) and authenticated by transcriptomics analysis, where gene expression differences between original N2a cells and prion-susceptible and -resistant subclones were investigated and documented (Marbiah et al., EMBO J 2014 Jul 17;33(14):1527-47; doi: 10.15252/embj.201387150).

Mycoplasma contamination

All actively used N2a sublines are tested for mycoplasma contamination and were confirmed negative. Regular mycoplasma screens are conducted in the tissue culture facility at least once every 2 years. No mycoplasma contaminations have been reported in the facility in the past 10 years.

Commonly misidentified lines
(See ICLAC register)

N/A

Animals and other research organisms

Policy information about studies involving animals; ARRIVE guidelines recommended for reporting animal research, and Sex and Gender in Research

Laboratory animals	Inbred male and female mice (strain: FVB/N) were required to setup breeding pairs for the preparation of primary neuronal cultures from embryonic e17 mouse brains as described in Methods.
Wild animals	Not applicable
Reporting on sex	Mice were used in this manuscript solely for the setup of breeding pairs and included male and female mice.
Field-collected samples	Not applicable
Ethics oversight	All procedures involving animals were performed under approval and license granted by the UK Home Office (Animals (Scientific Procedures) Act 1986), project license number 70/9022 in compliance with UCL institutional guidelines and Animal Research: Reporting of In Vivo Experiments (ARRIVE) guidelines.

Note that full information on the approval of the study protocol must also be provided in the manuscript.
